# Uterine Artery Doppler Indices as Predictor of Adverse Fetal Outcome in Hypertensive Disorders of Pregnancy: An Observational Study

**DOI:** 10.7759/cureus.49265

**Published:** 2023-11-22

**Authors:** Sakshi Duragkar, Kalyani S Mahajan, Deepika Dewani

**Affiliations:** 1 Obstetrics and Gynecology, Jawaharlal Nehru Medical College, Datta Meghe Institute of Higher Education and Research, Wardha, IND; 2 Obstetrics and Gynecology, Symbiosis Medical College for Women, Symbiosis International University, Pune, IND

**Keywords:** singleton pregnancy, pulsatility index (pi), resistance index (ri), systolic/diastolic (s/d) ratio, adverse fetal outcomes, uterine artery doppler, hypertensive disorders of pregnancy (hdp)

## Abstract

Background

Hypertensive disorders of pregnancy (HDP) pose significant risks to maternal and fetal health. The utility of Doppler indices in predicting adverse fetal outcomes in HDP patients remains an area of active research. This observational study aimed to assess the correlation between abnormal uterine artery Doppler indices and adverse fetal outcomes in HDP patients.

Methods

Over a two-year period, we enrolled 138 pregnant women with HDP beyond 28 weeks of gestation and singleton pregnancies. Detailed clinical assessments, laboratory investigations, and Doppler studies of the uterine artery were conducted. The Doppler indices that were assessed included the systolic/diastolic (S/D) ratio, resistance index (RI), and pulsatility index (PI). Adverse fetal outcomes were classified based on appearance, pulse, grimace, activity, and respiration (APGAR) scores, birth weight, NICU admissions, and perinatal deaths. Statistical analyses were performed to evaluate the predictive value of Doppler indices.

Results

Abnormal uterine artery Doppler indices, specifically an elevated S/D ratio and the presence of a diastolic notch showed a positive correlation with adverse fetal outcomes. However, Doppler indices such as PI and RI did not demonstrate a significant correlation with adverse fetal outcomes in HDP patients. These findings suggest that the S/D ratio and the presence of a diastolic notch in uterine artery Doppler studies hold potential as predictive markers for adverse fetal outcomes in HDP patients.

Conclusion

Uterine artery Doppler indices, specifically the S/D ratio and the presence of a diastolic notch, appear to be valuable predictors for adverse fetal outcomes in patients with hypertensive disorders of pregnancy. These findings underscore the importance of regular monitoring of uterine artery Doppler flow in the management of HDP to identify pregnancies at higher risk for adverse fetal outcomes.

## Introduction

Hypertensive disorders of pregnancy (HDP) encompass a spectrum of conditions that pose a substantial threat to both maternal and fetal health, making them a major concern in the field of obstetrics. These disorders, which include gestational hypertension, preeclampsia, and eclampsia, are characterized by elevated blood pressure levels during pregnancy and can lead to a range of complications, including maternal organ dysfunction and adverse fetal outcomes [1-2].

Among the various diagnostic tools employed to assess the severity and potential consequences of HDP, uterine artery Doppler ultrasound has emerged as a valuable and non-invasive technique. Uterine artery Doppler provides valuable insights into uteroplacental circulation, allowing clinicians to monitor blood flow resistance within the uterine arteries [2-4]. The uterine artery Doppler indices, such as the systolic-to-diastolic (S/D) ratio, pulsatility index (PI), and resistance index (RI), can be used to assess the impedance of blood flow in these vessels. Abnormalities in these indices have been associated with compromised uteroplacental perfusion, which, in turn, can result in adverse fetal outcomes [5-7].

This observational study seeks to investigate the utility of uterine artery Doppler indices as predictors of adverse fetal outcomes in patients with hypertensive disorders of pregnancy. It aims to explore the relationship between abnormal uterine artery Doppler findings and key fetal outcomes, including low birth weight (LBW) infants, neonates with Apgar scores less than 7, the necessity for neonatal intensive care unit (NICU) admission, and fetal mortality [8].

Understanding the potential of uterine artery Doppler as a predictive tool for adverse fetal outcomes in HDP is paramount for obstetric care. Such knowledge can guide healthcare providers in identifying high-risk pregnancies early on, enabling them to implement appropriate interventions and closely monitor pregnancies at risk. Ultimately, the findings of this study may contribute to improved maternal and fetal outcomes in the challenging landscape of hypertensive disorders of pregnancy.

## Materials and methods

Study design and location

This research employed an observational design and was conducted at the Department of Obstetrics and Gynecology, situated within Acharya Vinoba Bhave Rural Hospital (AVBRH) in Sawangi (Meghe), Wardha, Maharashtra, India, with ethical committee permission IEC/2020/9059. The study extended from September 2019 to August 2021 (Two years).

Study population

A purposive sampling technique was employed in this study; 138 pregnant women were diagnosed with hypertensive disorders of pregnancy, each carrying a singleton pregnancy with a gestational age surpassing 28 weeks and displaying abnormal Doppler studies, by the established inclusion and exclusion criteria.

Data collection

Data collection commenced with the acquisition of written informed consent from participants, which was conducted in the local language, Marathi. The recorded consents adhered to a structured proforma that was carefully designed and pre-tested. This proforma encompassed comprehensive information about each participant's medical history, clinical examinations, and ultrasonography. Inclusion criteria were specific to pregnant women with hypertensive disorders of pregnancy beyond 28 weeks of gestation, carrying singleton pregnancies, and exhibiting abnormal Doppler parameter changes. Exclusion criteria, conversely, entailed pregnant women with additional medical complications, spanning renal disorders, liver disorders, diabetes mellitus, heart disease, autoimmune disorders, chronic illnesses, multiple pregnancies, assisted conception, and congenital anomalies. Having provided informed consent, eligible pregnant women were recruited for the study. Moreover, demographic details were meticulously gathered utilizing the study proforma. A comprehensive assessment was conducted, involving thorough clinical and obstetrical examinations. Laboratory investigations were performed, adhering rigorously to established protocols for hypertensive disorders of pregnancy. Subsequently, obstetric ultrasound and color Doppler examinations were carried out at the department of radiodiagnosis, utilizing the ALOKA HITACHI ARIETTA 65 - G3003853 ultrasound unit. Key Doppler indices for the uterine artery, including the systolic/diastolic (S/D) ratio, resistance index (RI), and pulsatility index (PI), were specifically evaluated as part of this examination.

Technical Considerations

Several technical considerations played a pivotal role throughout the Doppler examination process. Firstly, the insonation angle was maintained at less than 60 degrees to ensure precise measurements. The measurement of pre-systolic and end-diastolic velocities was performed manually to guarantee accuracy. Doppler measurements were taken while the pregnant mother was recumbent during fetal inactivity and apnea. Additionally, the calculation of indices, such as the S/D ratio, RI, and PI, was conducted utilizing the relevant formulas, ensuring the integrity of the data.

Uterine Artery

The uterine artery was evaluated in the vicinity of the cervico-corporal junction. Doppler velocimetry measurements were collected, and the identification of abnormal findings included the presence of an early diastolic notch, an S/D ratio surpassing 2.6, or PI and RI values exceeding the 95th percentile.

Follow-Up Doppler Studies

Follow-up Doppler studies were carried out to monitor fetal well-being effectively as needed. The results analyzed for the study were derived from the most recent Doppler ultrasound conducted within one week of delivery. The management of cases was subsequently determined based on a careful assessment of clinical status and Doppler ultrasound reports, and pregnancy terminations were undertaken when deemed necessary. Patients were meticulously followed up until delivery, during which time fetal outcomes were closely scrutinized.

Adverse Fetal Outcome

The categorization of adverse fetal outcomes hinged on several key variables, including the APGAR score at five minutes, birth weight, NICU admissions, and perinatal death. Specifically, all neonates who exhibited an APGAR score below seven at five minutes, possessed a birth weight less than 2.5 kg, necessitated NICU admissions, or experienced perinatal deaths were classified as having suffered adverse fetal outcomes.

Sample size calculation

The research was conducted at a tertiary care institute in a rural setting. The incidence of hypertensive disorders of pregnancy (HDP) within the geographic region of the study site was determined to be within the range of 9-15% [9]. Based on this information and considering an assumed incidence of 10%, along with a 5% type I error (represented by the value of Z_(1-∝/2) set at 1.96). The required sample size was calculated to be 138 using these parameters.

Ethical clearance

Ethical clearance for the study was obtained from the Ethical Review Committee of Datta Meghe University of Medical Sciences, Wardha, with reference (IEC/2020/9059). Informed written consent was obtained from all eligible study subjects after explaining the methodology and relevance of the study in detail.

Data analysis

Data were analyzed using SPSS version 24.0 (IBM Inc. Armonk, New York). Qualitative data were expressed as percentages, and quantitative data were presented as mean ± SD and range values. Chi-square or Fisher's exact test was used for analyzing the relationship between qualitative data, while an independent sample 't' test was employed for quantitative data. Sensitivity, specificity, positive predictive value, negative predictive value, and diagnostic accuracy were calculated. A p-value less than 0.05 was considered statistically significant.

## Results

Table 1 illustrates the distribution of patients according to various demographic variables in the context of hypertensive disorders of pregnancy (HDP) with Doppler changes. Regarding age group, most patients fell within the 20-24 years bracket, accounting for 43.5% of the total, while patients aged 25-29 made up 34.1%. As assessed using the modified BG Prasad scale, the socioeconomic status revealed that 47.1% of participants belonged to the lower-class category. Among the HDP diagnoses, preeclampsia was the most common, representing 53.6% of patients, while other categories had minimal representation. In the context of parity, a significant proportion were primigravida, making up 67.4%. Lastly, regarding gestational age at delivery, the highest percentage of patients (51.5%) fell in the 33-36.6 weeks range.

**Table 1:** Distribution of patients based on the demographic variable in HDP with Doppler changes HDP - hypertensive disorders of pregnancy

Age group	No of patients	Percentage
≤19 years	4	2.9
20-24 years	60	43.5
25-29 years	47	34.1
30-35 years	24	17.4
35 years and above	3	2.2
Total	138	100.0
Socioeconomic status (modified BG Prasad)		
Lower-class	65	47.10
Lower-middle class	51	36.95
Middle class	16	11.62
Upper-middle class	4	2.89
Upper class	2	1.44
Total	138	100.0
Hypertensive disorder of pregnancy		
Gestational hypertension	57	41.3
Chronic hypertension	0	0.0
Preeclampsia	74	53.6
Eclampsia	7	5.1
Chronic hypertension with superimposed preeclampsia	0	0.0
Total	138	100.0
Parity		
Primigravida	93	67.4
Multigravida	45	32.6
Total	138	100.0
Gestational age at delivery		
28-32.6 weeks	22	15.9
33-36.6 weeks	71	51.5
37-40 weeks	45	32.6
Total	138	100.0

Table 2 presents the distribution of uterine artery Doppler indices among patients with hypertensive disorders of pregnancy (HDP). These indices include the S/D ratio, PI, RI, and the presence or absence of a notch in the Doppler waveform. For the S/D Ratio, 60.2% of patients displayed a normal ratio, while 39.8% had an abnormal ratio. Regarding PI, 44.21% showed a normal value, and 55.79% had an abnormal PI. Regarding RI, 34.79% of patients had a normal RI, and 65.21% exhibited an abnormal RI. Notably, regarding the presence of a notch in the Doppler waveform, 69.85% of patients had an absent notch , and 31.15% had a present notch.

**Table 2:** Distribution of uterine artery Doppler indices in HDP S/D - systolic/diastolic; PI - pulsatility index; RI - resistance index; HDP - hypertensive disorders of pregnancy

Uterine artery parameters		No of patients	Percentage
S/D ratio	Normal	83	60.2
	Abnormal	55	39.8
PI	Normal	61	44.21
	Abnormal	77	55.79
RI	Normal	48	34.79
	Abnormal	90	65.21
Notch	Absent	95	69.85
	Present	43	31.15

Table 3 summarizes patient distribution in hypertensive disorders of pregnancy (HDP) with Doppler changes. It categorizes patients based on the type of labor, mode of delivery, APGAR scores at five minutes, and birth weight. Most patients underwent induced labor and had lower segment cesarean section (LSCS) deliveries. Most newborns had APGAR scores greater than seven at five minutes, and the birth weight distribution varied, with a significant percentage falling within the low birth weight (LBW) range. 

**Table 3:** Distribution of patients based on delivery and fetal outcome details in HDP with Doppler changes HDP - hypertensive disorders of pregnancy; NVD - normal vaginal delivery; LSCS - lower segment Caesarean section; ELBW - extremely low birth weight; VLBW - very low birth weight; LBW - low birth weight

Variables	No of patients	Percentage
Type of labor		
Spontaneous	49	35.5
Induced	89	64.5
Total	138	100
Mode of delivery		
NVD	57	41.3
Instrumental delivery	8	5.8
LSCS	73	52.9
Total	138	100
APGAR score at five min		
0-6	71	51.4
>7	67	48.6
Total	138	100
Birth weight		
ELBW (<1 kg)	11	7.9
VLBW (1-1.5kg)	17	12.4
LBW (1.5-2.5kg)	59	42.8
Normal (>2.5kg)	51	36.9
Total	138	100

Table 4 displays the relationship between abnormal uterine artery Doppler indices and adverse fetal outcomes in hypertensive disorders of pregnancy (HDP). It shows that abnormal S/D ratios and the presence of a notch in the uterine artery are significantly associated with adverse outcomes. However, PI and RI do not exhibit such associations. Table 5 further explores this connection, revealing that abnormal uterine artery Doppler is linked to higher rates of NICU admissions and low birth weight infants. In Table 6, the accuracy of these Doppler indices in predicting adverse fetal outcomes is assessed, with the S/D ratio demonstrating the highest diagnostic accuracy. 

**Table 4:** Association between abnormal uterine artery Doppler indices with adverse fetal outcome in HDP S/D - systolic/diastolic; PI - pulsatility index; RI - resistance index; HDP - hypertensive disorders of pregnancy; S - significant; NS - not significant

Uterine artery parameters		Adverse outcome	Good outcome	X^2^	p-value
S/D ratio	Abnormal	40	15	31.83	0.01, S
	Normal	20	63		
PI	Abnormal	20	57	0.76	0.38, NS
	Normal	20	41		
RI	Abnormal	25	65	0.79	0.37, NS
	Normal	10	38		
Notch	Abnormal	27	16	25.89	0.01, S
	Normal	18	77		

**Table 5:** Co-relation of uterine artery Doppler with adverse fetal outcomes in HDP One neonate may show multiple outcomes LBW - low birth weight; HDP - hypertensive disorders of pregnancy; NS - not significant; S - significant

Fetal outcomes	Uterine artery				p-value
	Abnormal (N=53)	%	Normal (N=85)	%	
APGAR	19	35.84	22	25.88	0.21, NS
NICU	22	41.4	18	21.17	0.01, S
LBW	31	58.49	4	4.7	<0.01, S
Mortality	1	1.88	4	4.7	0.38, NS

**Table 6:** Accuracy of abnormal uterine artery doppler indices in anticipating adverse fetal outcome in HDP S/D - systolic/diastolic; PI - pulsatility index; RI - resistance index; HDP - hypertensive disorders of pregnancy; PPV - positive predictive value; NPV - negative predictive value; DA - diagnostic accuracy

Artery	Parameter	Sensitivity	Specificity	PPV	NPV	DA
Uterine artery	S/D	66.67	80.77	72.73	75.90	74.64
	PI	50	41.84	25.97	67.21	44.20
	RI	50	26.14	27.78	47.92	34.78
	Notch	64	87.50	74.42	81.05	75.36

## Discussion

Doppler ultrasound has played a significant role in comprehending uteroplacental and fetoplacental circulation in the context of hypertensive disorders of pregnancy (HDP), a subject extensively investigated by various researchers. In uncomplicated pregnancies, uteroplacental and fetoplacental circulation typically exhibit low resistance. However, HDP is characterized by deficient trophoblastic invasion, leading to aberrant placental vascular flow. Doppler studies play a pivotal role in identifying abnormal vascular resistance patterns in compromised fetuses, enabling timely intervention.

The incidence of hypertensive disorders of pregnancy (HDP) displays regional variability, and in the Indian context, reported rates typically range from 6% to 8%. However, our current study reveals a higher incidence, with 10% of the 138 diagnosed with HDP. Among these cases, 53.6% presented with preeclampsia, 41.3% with gestational hypertension, and 5.1% with eclampsia. Notably, our study did not identify any patients with chronic hypertension or chronic hypertension with superimposed preeclampsia. This elevated incidence in our findings prompts further exploration and consideration of potential contributing factors specific to our study population. Notably, the distribution of HDP subtypes in our study differs from other investigations. For instance, the absence of cases with chronic hypertension or chronic hypertension with superimposed preeclampsia sets our findings apart. Comparisons with existing studies, including those by Panda et al. (2021) [4], Sachdeva et al. (2011) [5], and Vidyadhar et al. (2011) [6], highlight the varied incidence rates and distributions of HDP reported in the literature. This observed heterogeneity underscores the complexity of HDP epidemiology, potentially influenced by regional, genetic, or environmental factors. Future research may benefit from exploring these factors to better understand the nuances of HDP prevalence and presentation.

Within the confines of the present study, 43.5% of hypertensive patients were within the age group of 20-24 years, while only 2.1% belonged to the age group exceeding 35 years. This distribution aligns with findings in studies by Parmar et al. (2017) [7], Bairwa et al. (2020) [8], Gaikwad et al. (2017) [9], and Mishra et al. (2020) [10], all of which reported similar age distributions among hypertensive pregnant women. Low socioeconomic status has been associated with nutritional challenges, reduced antenatal care, and unsanitary hygienic conditions, as highlighted in a multinational population-level analysis by Magee et al. (2019) [11]. In the current study, most HDP patients (47.1%) belonged to the lower socioeconomic class, with 37.0% hailing from the lower middle class. These findings are consistent with Lakhute et al. (2021) [12], who asserted that hypertension during pregnancy is more prevalent among those in lower socioeconomic strata.

A significant proportion (92.1%) of patients in the current study resided in rural areas, a trend consistent with the study conducted by Bairwa et al. (2020) [8], where 70% of hypertensive pregnant women were from rural regions. In the present study, 83.3% of patients had a normal body mass index (BMI). Studies by Bener et al. (2013) [13] and Ehrenthal et al. (2010) [14] have shown that a higher BMI increases the risk of HDP. However, the mean BMI in the present study was higher than that reported by Parmar et al. (2020) [7].

A majority (67.4%) of hypertensive pregnancy patients in the present study were primigravida, a pattern consistent with findings from Sibai et al. (2005) [15], Bairwa R et al. (2020) [8], and Gaikwad et al. (2017) [9], suggesting that primigravida is a risk factor for hypertension during pregnancy. More than half (51.5%) of the patients in the present study delivered before 37 weeks of gestation, with an average gestational age of 37.21 weeks. These findings align with the study by Buchbinder et al. (2002) [16] and the study by Parmar et al. (2017) [7], but differ from the study by Bairwa et al. (2020) [8], which reported mostly term deliveries. In cases of HDP, insufficient trophoblastic invasion leads to increased resistance in spiral arteries, obstructing blood flow in uterine arteries.

Within the present study, 39.8% of patients exhibited an abnormal uterine artery S/D ratio, consistent with the findings in the study conducted by Nagar et al. (2015) [17] and Fleischer (1986) [18] but in contrast with the results of Konwar et al. (2021) [19], which indicated a higher incidence of abnormality in the S/D ratio. Abnormal uterine artery PI and RI were observed in 55.79% and 65.21% of patients, respectively. The mean PI and RI in the present study aligned with those reported by Parmar et al. (2020) [7]. HDP is associated with adverse fetal outcomes due to compromised uteroplacental and fetoplacental circulation. In the present study, most (54.8%) of patients delivered full-term babies, while 45.2% delivered preterm. The mean birth weight in the present study was 2.47 kg, consistent with the findings in studies by Parmar et al. (2017) [7] and Patel et al. (2019) [20], which also reported low birth weights.

Limitation

One of the limitations of this study is the relatively small sample size. A larger sample size could provide more statistical power and potentially reveal additional correlations between uterine artery Doppler indices and adverse fetal outcomes. This research was conducted in a single center, which may limit the generalizability of the findings. Multicenter studies involving diverse populations could provide more comprehensive insights into the topic.

## Conclusions

In conclusion, our study reveals that abnormal uterine artery Doppler indices, specifically the S/D ratio and the presence of a diastolic notch in the waveform, are valuable predictors for adverse fetal outcomes in patients with hypertensive disorders of pregnancy. However, Doppler indices such as PI and RI did not demonstrate a significant correlation with adverse fetal outcomes in this patient population. These findings underscore the importance of regular monitoring of uterine artery Doppler flow in the management of hypertensive disorders of pregnancy, as it can help identify pregnancies at higher risk for adverse fetal outcomes.
